# The Role of Glymphatic Clearance in Affective Decision‐Making in Adolescents with Non‐Suicidal Self‐Injury

**DOI:** 10.1002/cns.71159

**Published:** 2026-10-01

**Authors:** Hongping Wang, Jiahua Zhang, Ruizi Pan, Xiaoqian Ye, Rong Wang, Ke Lv, Yue Yu, Qianqian Li, Ting Zhang, Dongpeng Wu, Dai Zhang, Yanghua Tian

**Affiliations:** ^1^ Department of Neurology The Second Affiliated Hospital of Anhui Medical University Hefei China; ^2^ The School of Mental Health and Psychological Sciences Anhui Medical University Hefei China; ^3^ Department of Radiology The Second Affiliated Hospital of Anhui Medical University Hefei China; ^4^ Medical Imaging Research Center Anhui Medical University Hefei China; ^5^ Department of Neurology The First Affiliated Hospital of Anhui Medical University Hefei China; ^6^ Department of Psychology and Sleep Medicine The Second Affiliated Hospital of Anhui Medical University Hefei China; ^7^ Department of Psychiatry The First Affiliated Hospital of Anhui Medical University Hefei China; ^8^ Division of Life Sciences and Medicine, Department of Neurology, The First Affiliated Hospital of USTC University of Science and Technology of China Hefei China; ^9^ Anhui Province Key Laboratory of Cognition and Neuropsychiatric Disorders Hefei China; ^10^ Collaborative Innovation Center of Neuropsychiatric Disorders and Mental Health Hefei China

**Keywords:** affective decision‐making, gBOLD‐CSF coupling, glymphatic system, non‐suicidal self‐injury, sleep disturbance

## Abstract

**Background:**

Adolescents with non‐suicidal self‐injury (NSSI) frequently exhibit severe sleep disturbances and heterogeneous cognitive impairments, yet the underlying neurophysiological basis remains unclear. The glymphatic system, a sleep‐dependent macroscopic clearance network, is critical for metabolic waste removal and neurocognitive homeostasis. Therefore, this study aimed to investigate the physiological role of glymphatic function in linking sleep burden to distinct cognitive performances in adolescents with NSSI.

**Methods:**

We assessed 44 NSSI adolescents and 33 healthy controls (HCs). Using resting‐state functional magnetic resonance imaging (rs‐fMRI), glymphatic function was quantified by calculating the coupling strength between global blood‐oxygen‐level‐dependent (gBOLD) signals and cerebrospinal fluid (CSF) flow. To capture the heterogeneity in their cognitive profiles, we evaluated “hot” affective decision‐making via the Iowa Gambling Task (IGT) and “cold” executive function via the Wisconsin Card Sorting Test (WCST). Subjective sleep quality was assessed using the Pittsburgh Sleep Quality Index (PSQI).

**Results:**

In the NSSI group, poorer sleep quality was positively correlated with stronger gBOLD‐CSF coupling (*r* = 0.317, *p* = 0.036), and stronger gBOLD‐CSF coupling predicted better IGT performance, especially during the high‐load strategy phase (Block 3) (*r* = 0.447, *p* = 0.002). Furthermore, glymphatic function fully mediated the relationship between sleep quality and affective decision‐making performance (indirect effect = 0.682, 95% CI [0.062, 1.515]). Interaction analyses confirmed this physiological‐behavioral coupling was disease‐specific (present only in NSSI; interaction *p* = 0.011) and domain‐specific (supporting “hot” affective cognition rather than “cold” executive function).

**Conclusions:**

In NSSI adolescents, sleep‐associated glymphatic upregulation reflects a stress‐induced compensatory response. This mechanism domain‐specifically preserves high‐load affective decision‐making against severe psychopathological stress. These findings highlight brain metabolic clearance as a novel physiological target for bolstering cognitive resilience in high‐risk youth.

## Graphical abstract

In adolescents with non‐suicidal self‐injury (NSSI), characterized by sleep disturbances and metabolic burden, a disease‐specific, compensatory up‐regulation of glymphatic function (stronger gBOLD‐CSF coupling) selectively supports “hot” (affective) cognition while leaving “cold” (executive) cognition unlinked, functioning as a pathological buffer.

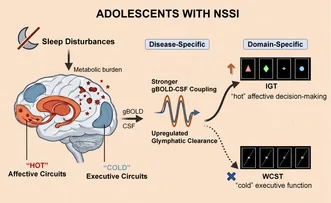

## 1 Introduction

Non‐suicidal self‐injury (NSSI) refers to the intentional and recurrent infliction of damage to one's own body in the absence of suicidal intent [1]. Global meta‐analyses reveal the alarming scale of this issue, estimating that over 22% of the general youth population engages in such behaviors [2]. Consequently, NSSI has emerged as one of the most formidable clinical challenges in contemporary adolescent psychiatry [3, 4]. Beyond immediate somatic injury, NSSI is closely linked to a spectrum of adverse clinical outcomes, notably cognitive impairments, sleep dysregulation, and heightened impulsivity [5, 6, 7]. However, current behavioral studies regarding cognitive and decision‐making functions in NSSI yield markedly inconsistent results. While some studies report widespread cognitive deficits in executive control and affective decision‐making [8, 9], others find that many NSSI adolescents perform normally on specific cognitive tasks [10, 11]. This high degree of behavioral heterogeneity suggests that relying solely on overarching cognitive phenotyping is inherently limited, highlighting the need to investigate the underlying neurophysiological metrics.

To decode this heterogeneity, it is crucial to recognize that cognitive processing is not monolithic; it can be broadly dissociated into “hot” affective decision‐making and “cold” executive function [12, 13]. “Hot” cognition involves emotion‐ and reward‐laden processing governed heavily by the limbic‐striatal circuitry, whereas “cold” cognition relies on the dorsolateral prefrontal cortex for emotionally neutral, logic‐based problem solving [14]. In the NSSI population, the limbic‐striatal networks governing emotion regulation and “hot” cognition are specifically targeted and chronically hyperactive [15, 16]. Sustaining such highly activated cognitive networks requires extremely efficient cerebral metabolic homeostasis [17]. This chronic hyperactivation generates a substantial metabolic burden, thereby rendering these specific “hot” circuits highly dependent on efficient metabolic clearance. The glymphatic system, which serves as the brain's primary paravascular metabolic clearance pathway, has been established as a critical physiological mechanism for maintaining this neurophysiological and cognitive health [18, 19]. Crucially, glymphatic function is intimately tied to sleep quality, as sleep acts as the primary driver of cerebral metabolic clearance [20]. Given that severe sleep disturbances are a quintessential clinical feature of the NSSI cohort [21, 22], it remains an unanswered question how such extreme sleep deprivation reshapes glymphatic clearance dynamics, and how these physiological alterations subsequently map onto domain‐specific cognitive performance.

Measuring glymphatic activity in living humans has traditionally been difficult. However, recent neuroimaging advances offer a non‐invasive solution by tracking the coupling between the global blood‐oxygen‐level‐dependent (gBOLD) signal and cerebrospinal fluid (CSF) flow [23]. The physiological mechanism behind this metric is straightforward. During sleep or resting states, slow neural waves cause natural drops in cerebral blood volume [24]. This reduction acts like a physiological piston, drawing CSF into the brain tissue to wash away metabolic waste [23, 25]. By capturing this dynamic process, gBOLD‐CSF coupling serves as an effective, observable marker of the brain's clearance capacity. It has become a valuable tool for linking sleep quality with cognitive performance across various psychiatric conditions [26, 27, 28].

To decipher the physiological mechanisms underlying cognitive heterogeneity in self‐injuring youth, we quantified glymphatic function in NSSI adolescents using gBOLD‐CSF coupling. We specifically aimed to clarify how glymphatic integrity differentially impacts “hot” affective decision‐making versus “cold” executive function. Given the intrinsic link between sleep and metabolic clearance, we further hypothesized that glymphatic function mediates the relationship between severe sleep disturbances and specific cognitive outcomes. Ultimately, this study aims to reveal how sleep‐driven physiological alterations map onto the complex behavioral profiles of this vulnerable population.

## 2 Materials and Methods

### 2.1 Participants

Adolescents with NSSI were consecutively recruited from the Second Affiliated Hospital of Anhui Medical University. Inclusion criteria were: (1) primary diagnosis of NSSI according to DSM‐5; (2) sufficient cognitive capacity to complete assessments; and (3) provision of informed consent. Exclusion criteria included: acute psychosis or suicidality, pervasive developmental disorders, organic brain diseases, unstable medical conditions, or MRI contraindications (e.g., claustrophobia, metallic implants). Concurrently, healthy controls (HCs) with no lifetime history of psychiatric diagnoses or NSSI behaviors were recruited via community advertisements.

### 2.2 Neuropsychological Assessment

All participants underwent a comprehensive neuropsychological battery administered by trained psychiatrists. The characteristics of self‐injurious behaviors were evaluated using the Adolescent Non‐Suicidal Self‐Injury Assessment Questionnaire (ANSAQ) and the Clinician‐Rated Severity of Non‐Suicidal Self‐Injury (CRSNSSI) [29, 30]. These instruments were used to comprehensively measure the frequency and overall severity of NSSI behaviors over the past year. To quantify the severity of emotional dysregulation, the 17‐item Hamilton Depression Rating Scale (HAMD‐17) was employed to assess depressive symptoms [31]. The Pittsburgh Sleep Quality Index was administered to evaluate subjective sleep quality over the preceding month [32]. Barratt Impulsiveness Scale 11 Chinese Revised Version (BIS‐11) is composed of three subscales: cognitive impulsiveness, motor impulsiveness, and non‐planning impulsiveness [33]. Each subscale has scores ranging from 10 to 50, where higher scores denote increased impulsivity. Specifically, the total score and subscales were analyzed to distinguish stable personality traits from state‐dependent cognitive decision‐making deficits. These clinician‐rated scales served to establish the baseline of clinical burden in the NSSI group.

To comprehensively assess cognitive profiles across both affective and executive domains, two distinct paradigms were utilized. This approach allowed for a comparative analysis of how brain clearance relates to emotion‐based versus logic‐based processing. Iowa Gambling Task (IGT): Used to assess “hot” affective decision‐making under ambiguity. The net score (calculated as the number of advantageous choices minus disadvantageous choices) and the block‐by‐block learning curve were used as primary indices of reward‐based decision‐making strategies [34, 35]. Wisconsin Card Sorting Test (WCST): Used to assess “cold” executive function, specifically cognitive flexibility and set‐shifting capabilities [36]. Total errors and perseverative errors were recorded to index the integrity of the dorsolateral prefrontal cortex‐mediated executive control [37, 38], serving as a contrast to the affective processing measured by the IGT.

### 2.3 Image Acquisition

MRI data were acquired using a 3.0‐T Discovery GE750 MR scanner (GE Healthcare, Buckinghamshire, UK) equipped with an 8‐channel head coil. Foam padding and earplugs were employed to restrict head motion and attenuate noise. We acquired high‐resolution T1‐weighted anatomical images (TR/TE = 8.16/3.18 ms, TI = 450 ms, flip angle = 12°, FOV = 256 × 256 mm^2^, matrix = 256 × 256) consisting of 188 sagittal slices with an isotropic voxel size of 1 mm^3^. Data quality was verified by two experimenters to confirm full brain coverage. For functional MRI, a total of 217 echo‐planar imaging (EPI) volumes were collected. Acquisition parameters were: TR/TE = 2400/30 ms, flip angle = 90°, FOV = 192 × 192 mm^2^, and matrix = 64 × 64. Each volume contained 46 slices (thickness = 3 mm), resulting in a voxel size of 3 × 3 × 3 mm^3^. Continuous MR‐compatible video monitoring of open‐eye fixation ensured participant wakefulness, with the operator intervening to pause the session, re‐brief the participant, and repeat the scan should prolonged eye closure or drowsiness occur.

### 2.4 Data Preprocessing and gBOLD‐CSF Coupling Analysis

#### 2.4.1 Preprocessing

Preprocessing was performed using DPABI v6.0 (https://rfmri.org/dpabi) and SPM12 (https://www.fil.ion.ucl.ac.uk/spm/software/spm12/) implemented in MATLAB 2023b. We discarded the initial 10 volumes to ensure magnetization equilibrium. Subsequent steps included slice timing correction, motion realignment, linear trend removal, spatial smoothing (4‐mm FWHM kernel), and band‐pass filtering (0.01–0.1 Hz). Crucially, to preserve hemodynamic information essential for this specific analysis, we explicitly omitted the standard nuisance regression of global, CSF, and motion signals [23].

#### 2.4.2 Regions of Interest (ROI) Definition

Cortical gray matter ROIs were derived from the Harvard‐Oxford atlas. To prevent signal loss from registration, these ROIs were inverse‐transformed from MNI‐152 space to each subject's native space. CSF inflow ROIs were manually delineated on the basal fMRI slices near the cerebellum, where inflow effects are maximal [23]. ROI anatomical accuracy was verified via co‐registered T1 images (Figure 1).

**FIGURE 1:**
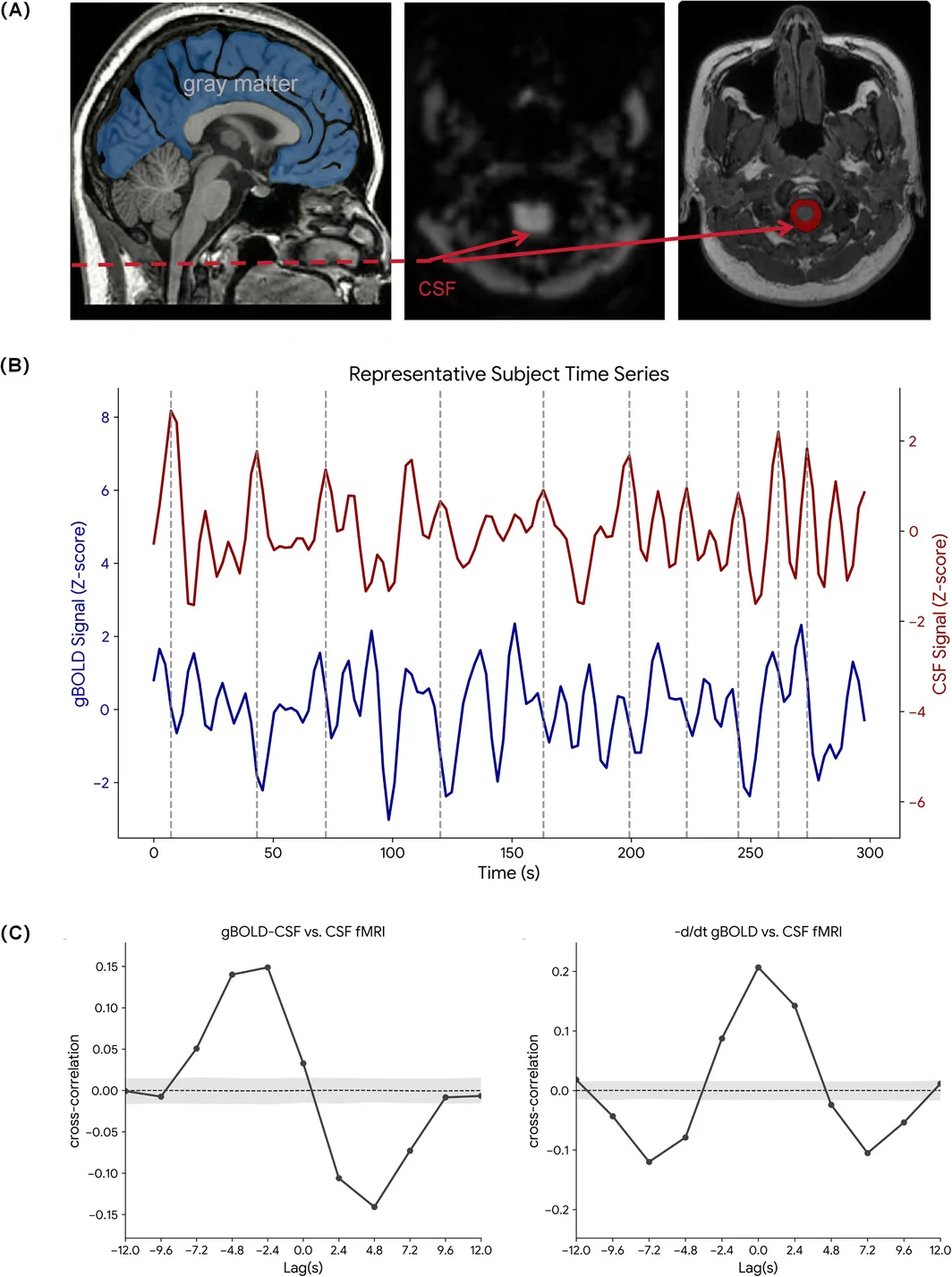
Methodological framework and validation of gBOLD‐CSF coupling. (A) Signal extraction framework. Left: Schematic of signal sources; the global BOLD signal (blue line) was extracted from the whole‐brain gray matter mask (blue overlay), while the CSF signal (red line) was derived from the bottom‐most slices. Middle: Representative slice from the resting‐state fMRI scan, showing the bottom‐most axial section used for CSF signal extraction. Right: Corresponding axial T1‐weighted structural image, with the CSF‐ROI highlighted in red. This region, located near the lower cerebellum and fourth ventricle, optimally captures CSF inflow effects. (B) Individual time series. Representative time series of the global BOLD signal (gBOLD, purple) and cerebrospinal fluid signal (CSF, red) from a single participant. The distinct anticorrelated pattern demonstrates that CSF inflow peaks align with the descending phase of the gBOLD signal (marked by dashed vertical lines). (C) Group‐level validation. Left: Group‐averaged cross‐correlation function between gBOLD and CSF signals across all participants. The curve exhibits a prominent negative peak at a lag of +4.8 s. Right: Cross‐correlation between the negative first derivative of the gBOLD signal and the CSF signal, showing a significant positive peak at zero lag. Gray dashed lines in (C) represent the 95% confidence intervals of the null distribution generated via permutation testing.

#### 2.4.3 gBOLD‐CSF Coupling Calculation

Signal extraction followed established protocols [39, 40]: CSF signals were taken from unsmoothed images, and gBOLD signals from unnormalized images. Time series were *Z*‐normalized and averaged within ROIs. We computed cross‐correlations in MATLAB using gBOLD as the reference across lags of ±12 s. Since BOLD oscillations precede CSF flow, manifesting as a strong anticorrelation at positive lags [41], we quantified coupling strength using the peak anticorrelation. To link cerebral blood volume oscillations to CSF inflow, we also correlated the negative gBOLD derivative with CSF signals. For interpretation, the coupling index was inverted so that higher positive values reflect a stronger association between global brain activity and CSF flow.

#### 2.4.4 Validation

We validated the coupling specificity using a permutation test; gBOLD and CSF time series were randomly shuffled across subjects 10,000 times to generate a null distribution. Statistical significance (*p*‐values) was determined by comparing the observed coupling index against this null distribution.

### 2.5 Statistical Analysis

Data analyses were conducted using SPSS v26.0 (IBM Corp., Armonk, NY) and Python 3.8 scripts (Scipy/Statsmodels). Continuous variables were assessed for normality using the Shapiro–Wilk test. Independent samples *t*‐tests were utilized for normally distributed data, whereas non‐parametric tests (e.g., Kruskal–Wallis *H* tests) were applied for variables violating normality.

#### 2.5.1 Demographic and Clinical Comparisons

Continuous variables (e.g., age, clinical scores, gBOLD‐CSF coupling) were compared using two‐sample *t*‐tests or Mann–Whitney *U* tests based on data distribution, while categorical variables (e.g., gender) were assessed using Chi‐square tests.

#### 2.5.2 Brain‐Behavior Associations

To investigate the clinical and cognitive significance of glymphatic function, partial correlation analyses were conducted within the NSSI group, controlling for age, gender, and mean framewise displacement (FD) to mitigate the potential confounding effects of demographic and head motion factors. Specifically, bivariate unadjusted correlations and subsequent partial Pearson correlations were used to examine the associations between the strength of gBOLD‐CSF coupling and continuous variables, including clinical burden and cognitive performance metrics. For the ordinal clinical variable (i.e., CRSNSSI grade), partial Spearman's rank correlation was applied. To account for multiple testing, the Benjamini‐Hochberg False Discovery Rate (FDR) procedure was applied separately to the primary cognitive outcome metrics and the supplementary clinical variables. Furthermore, to determine whether the relationship between glymphatic clearance and cognitive preservation was disease‐specific, a general linear model (GLM) was employed to test the Group × Coupling interaction effect on cognitive performance, adjusting for the same covariates.

#### 2.5.3 Subgroup and Exploratory Mediation Analyses

Given the high prevalence of sleep disturbances in our cohort, an extreme‐group approach was employed to isolate distinct clinical phenotypes and maximize physiological contrast [42]. Specifically, the NSSI cohort was stratified by PSQI tertiles—a mathematically established threshold for optimizing group differentiation [43]: the top (PSQI > 12) and bottom (PSQI ≤ 10) tertiles defined the NSSI‐High and NSSI‐Low subgroups, respectively. To rigorously rule out the confounding effect of micro‐head movements and pharmacological interventions on fluid dynamics, a Quade's non‐parametric analysis of covariance (rank‐based ANCOVA) was subsequently employed, adjusting strictly for mean FD and medication status.

We conducted a simple mediation analysis to explore the mechanistic links among sleep quality, glymphatic function, and decision‐making in the NSSI group. In this model, subjective sleep quality (PSQI score) was set as the independent variable. Affective decision‐making (IGT net score) served as the dependent variable. Glymphatic strength, defined as the inverted gBOLD‐CSF coupling magnitude, was used as the mediator. All analyses were performed using the Python Statsmodels module based on ordinary least squares (OLS) regression. We estimated three main pathways: the effect of the independent variable on the mediator (path a), the effect of the mediator on the dependent variable (path b), and the direct/total effects on the dependent variable (paths c′ and c). To test the significance of the indirect effect, we utilized a non‐parametric bootstrapping procedure with 5000 resamples. The mediation effect was considered statistically significant if the 95% confidence interval (CI) did not include zero. Statistical significance was set at two‐tailed *p* < 0.05.

## 3 Results

### 3.1 Validation of the gBOLD‐CSF Coupling

The group‐averaged cross‐correlation between gBOLD and CSF signals exhibited a biphasic pattern across both the entire cohort (*N* = 77) and the HC group (*N* = 33; Figure 1C, Left). Specifically, a significant positive peak emerged at a lag of −2.4 s (*r* = 0.15, *p* < 0.001), followed by a significant negative peak at a lag of +4.8 s (*r* = −0.15, *p* < 0.001). Furthermore, the cross‐correlation between the negative first derivative of the gBOLD signal and the CSF signal yielded a significant positive peak at zero lag (*r* = 0.14, *p* < 0.001; Figure 1C, Right) in both groups. Representative time series illustrated that CSF inflow peaks consistently coincided with the descending phase of the gBOLD signal (Figure 1B). Consequently, the coupling strength at the +4.8 s lag was utilized as the primary metric for all subsequent group comparisons.

### 3.2 Demographic and Clinical Characteristics

The demographic and clinical data of the 44 adolescents with NSSI and 33 HCs are summarized in Table 1. The two groups were well‐matched, showing no significant differences in age (*Z* = −1.15, *p* = 0.249), gender (*χ* ^2^ = 2.25, *p* = 0.134), education (*Z* = −1.73, *p* = 0.083), or mean FD (*Z* = −0.45, *p* = 0.654). Regarding clinical burden, the NSSI group presented significantly elevated symptom severity across mood and sleep domains. Compared to HCs, the patient group exhibited significantly higher depressive symptoms on the HAMD‐17 (*Z* = −7.56, *p* < 0.001) and reported significantly poorer subjective sleep quality on the PSQI (*t* = 9.03, *p* < 0.001). Within the patient cohort, the mean CRSNSSI score was 2.36 ± 1.24 (NSSI frequency distribution detailed in Table S1). In terms of trait impulsivity, the patient cohort scored significantly higher on the BIS‐11 total scale (*t* = 5.34, *p* < 0.001), primarily driven by the motor and non‐planning subscales (Table S2). Patient medication status is detailed in Table S3.

**TABLE 1:** Demographic and clinical characteristics.

Characteristic	NSSI (*n* = 44)	HC (*n* = 33)	Statistic (*t*/*Z*/*χ* ^2^)	*p*
*Demographics*				
Age (years)^a^	15 (15, 17)	16 (15, 17)	−1.15	0.249
Gender (male/female)^b^	6/38	10/23	2.25	0.134
Education (years)^a^	10 (9, 11)	11 (10, 11)	−1.73	0.083
Head motion (mean FD)^a^	0.09 (0.08, 0.12)	0.09 (0.07, 0.12)	−0.45	0.654
*Clinical symptoms*				
CRSNSSI^c^	2.36 ± 1.24	0.00 ± 0.00	12.64	< 0.001***
HAMD^a^	22.50 (19.75, 27.25)	0 (0, 2)	−7.56	< 0.001***
BIS‐11^c^	54.69 ± 11.75	42.32 ± 8.57	5.34	< 0.001***
PSQI^c^	11.09 ± 3.18	4.27 ± 3.35	9.03	< 0.001***
*Cognitive performance*				
IGT net score^a^	−1 (−8, 4)	2 (−10, 18)	−1.01	0.312
WCST total errors^a^	29.00 (23.75, 37.50)	33 (21, 41)	−0.22	0.829
*gBOLD‐CSF coupling*				
Negative coupling^c^	−0.15 ± 0.12	−0.15 ± 0.13	−0.08	0.935

*Note:* Data are presented as mean ± SD or median (25th, 75th percentiles).

Abbreviations: BIS‐11, Barratt Impulsiveness Scale‐11; CRSNSSI, Clinician‐Rated Severity of Non‐Suicidal Self‐Injury; FD, framewise displacement; gBOLD‐CSF, global blood‐oxygen‐level‐dependent signal to cerebrospinal fluid; HAMD, hamilton depression scale; HC, healthy control; IGT, Iowa gambling task; NSSI, non‐suicidal self‐injury; PSQI, Pittsburgh Sleep Quality Index; SD: standard deviation; WCST, Wisconsin Card Sorting Test.

^a^ Mann‐Whitney *U* test (*Z* values reported).

^b^ Chi‐square test.

^c^ Two‐sample *t*‐test.

*** *p* < 0.001.

### 3.3 Glymphatic Function Across Sleep Subgroups and Cognitive Performance

Subjective sleep quality differed significantly among the HC, Low PSQI, and High PSQI subgroups (*H* = 44.21, *p* < 0.001; Figure 2A). Overall negative gBOLD‐CSF coupling did not differ significantly between the entire NSSI cohort and HCs (*t* = −0.08, *p* = 0.935). When evaluating glymphatic coupling strength across the three subgroups, a rank‐based ANCOVA strictly controlling for both mean FD and medication status yielded a main effect of *F*_(2,64)_ = 2.13 (*p* = 0.128). To isolate the physiological contrast driven by severe sleep burden, post hoc pairwise comparisons were subsequently conducted under the same covariate‐adjusted framework. These analyses revealed a significantly greater glymphatic strength in the High PSQI subgroup than in the Low PSQI subgroup (*F* = 5.19, *p* = 0.030, Cohen's *d* = 0.684). In contrast, no significant differences were observed when comparing HCs to either the High PSQI subgroup (*F* = 1.66, *p* = 0.204, Cohen's *d* = 0.473) or the Low PSQI subgroup (*F* = 0.26, *p* = 0.611, Cohen's *d* = 0.161; Figure 2B). Higher PSQI scores were significantly correlated with stronger glymphatic coupling (*r* = 0.317, *p* = 0.036). Notably, this positive linear association remained robust after strictly controlling for baseline depressive severity via HAMD‐17 scores (*r*_partial_ = 0.321, *p* = 0.034; Figure S1A).

**FIGURE 2:**
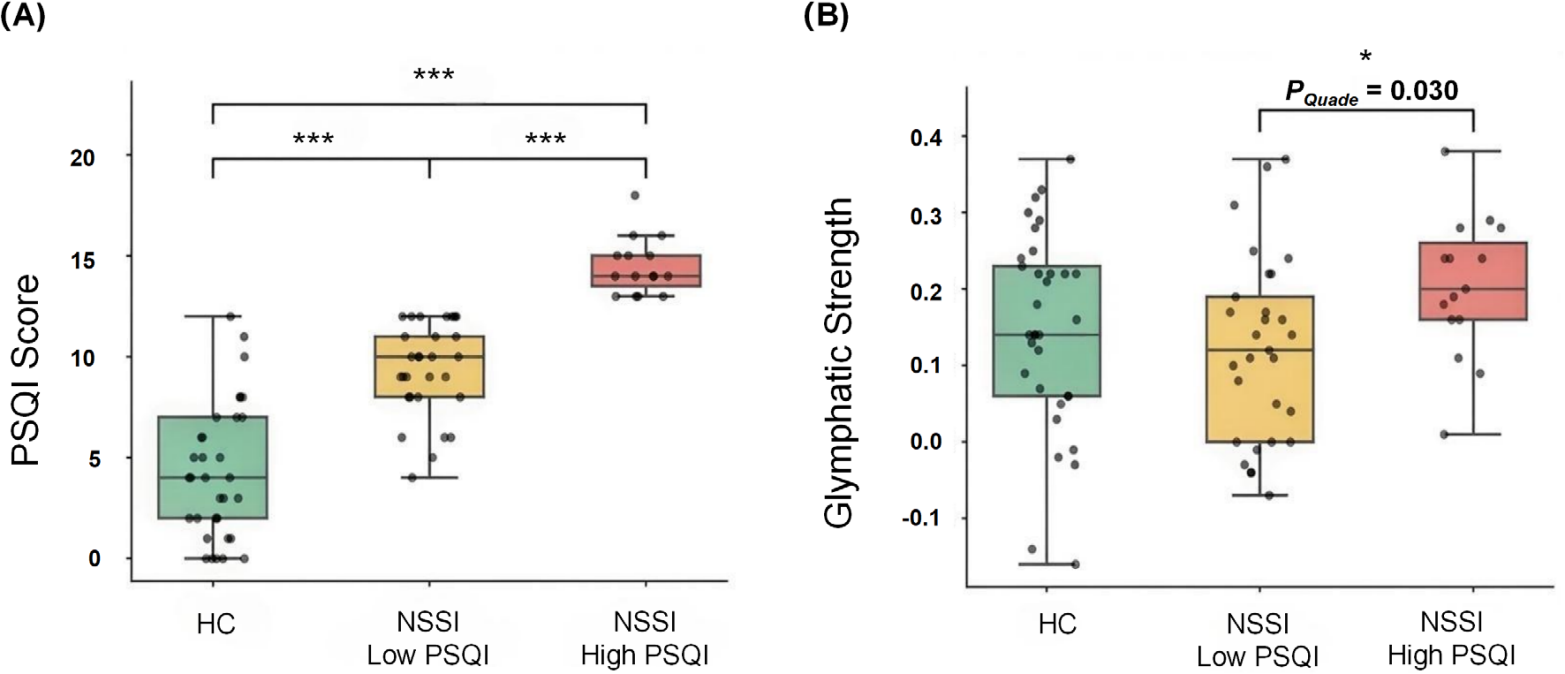
Comparisons of subjective sleep quality and glymphatic strength among HC and NSSI subgroups. The NSSI cohort was stratified into two subgroups based on the severity of sleep disturbances: NSSI Low PSQI (bottom 33.3% of scores) and NSSI High PSQI (top 33.3% of scores). (A) Comparison of PSQI scores across the three groups. (B) Comparison of glymphatic strength across the groups. The boxplots display the median and interquartile ranges, with overlaid scatter plots illustrating the underlying individual data distribution. In (B), to rigorously rule out the confounding effects of micro‐head movements and pharmacological interventions on fluid dynamics, a Quade's non‐parametric analysis of covariance (rank‐based ANCOVA) was employed for pairwise comparisons, adjusting for both mean FD and medication status. The significant physiological difference exclusively between the extreme sleep subgroups was robustly preserved (*p* = 0.030). *p < 0.05, ***p < 0.001. HC, healthy control; NSSI, non‐suicidal self‐injury; PSQI, Pittsburgh Sleep Quality Index.

Regarding cognitive performance, the NSSI group scored significantly lower than HCs on IGT Block 1 (*Z* = −2.29, *p* = 0.022; Table S4). Performance across IGT Blocks 2–5 and the overall IGT Net Score (*Z* = −1.01, *p* = 0.312) did not differ significantly between the two groups. WCST metrics showed no significant group differences across any of the assessed sub‐dimensions, including total errors (*Z* = −0.22, *p* = 0.829) and perseverative errors (*Z* = −0.52, *p* = 0.606; Table 1, Table S4).

### 3.4 Associations With Clinical Symptom Severity

Within the NSSI cohort, glymphatic coupling strength showed no significant correlations with HAMD, BIS‐11, or CRSNSSI in either unadjusted models or models adjusting for age, gender, and mean FD (all *p* > 0.05; Table S5).

### 3.5 Domain‐ and Disease‐Specific Associations of Glymphatic Function With Cognitive Performance

Within the NSSI cohort, stronger coupling corresponded to higher overall IGT Net Scores (*r*_partial_ = 0.402, *p* = 0.007; *p*_FDR_ = 0.032; Figure 3A) and elevated scores on IGT Block 3 (*r*_partial_ = 0.447, *p* = 0.002; *p*_FDR_ = 0.018; Figure 3B, Table S6). This specific relationship between glymphatic strength and IGT Block 3 performance was significantly moderated by group status (Group × Coupling interaction: *p* = 0.011; Figure 3B). Simple slope analyses demonstrated that stronger glymphatic coupling was robustly associated with higher Block 3 scores exclusively within the NSSI cohort (Slope = 17.3, *p* = 0.001), a relationship entirely absent in HCs (Slope = −8.8, *p* = 0.430). These functional associations remained highly significant even after additionally controlling for HAMD‐17 scores (IGT Net Score: *r*_partial_ = 0.402, *p* = 0.007; Figure S1B). Such correlational patterns did not extend to the remaining IGT blocks (all *p* > 0.05). Conversely, WCST variables yielded no significant associations, including total errors (*r*_partial_ = 0.270, *p* = 0.076), perseverative errors (*r*_partial_ = 0.195, *p* = 0.204), and categories completed (*r*_partial_ = −0.163, *p* = 0.291; Table S6). Furthermore, a formal moderation analysis confirmed that this specific physiological‐behavioral association was remarkably stable and not moderated by age (Interaction *p* = 0.769; Figure S2), indicating a consistent compensatory mechanism across the evaluated neurodevelopmental window.

**FIGURE 3:**
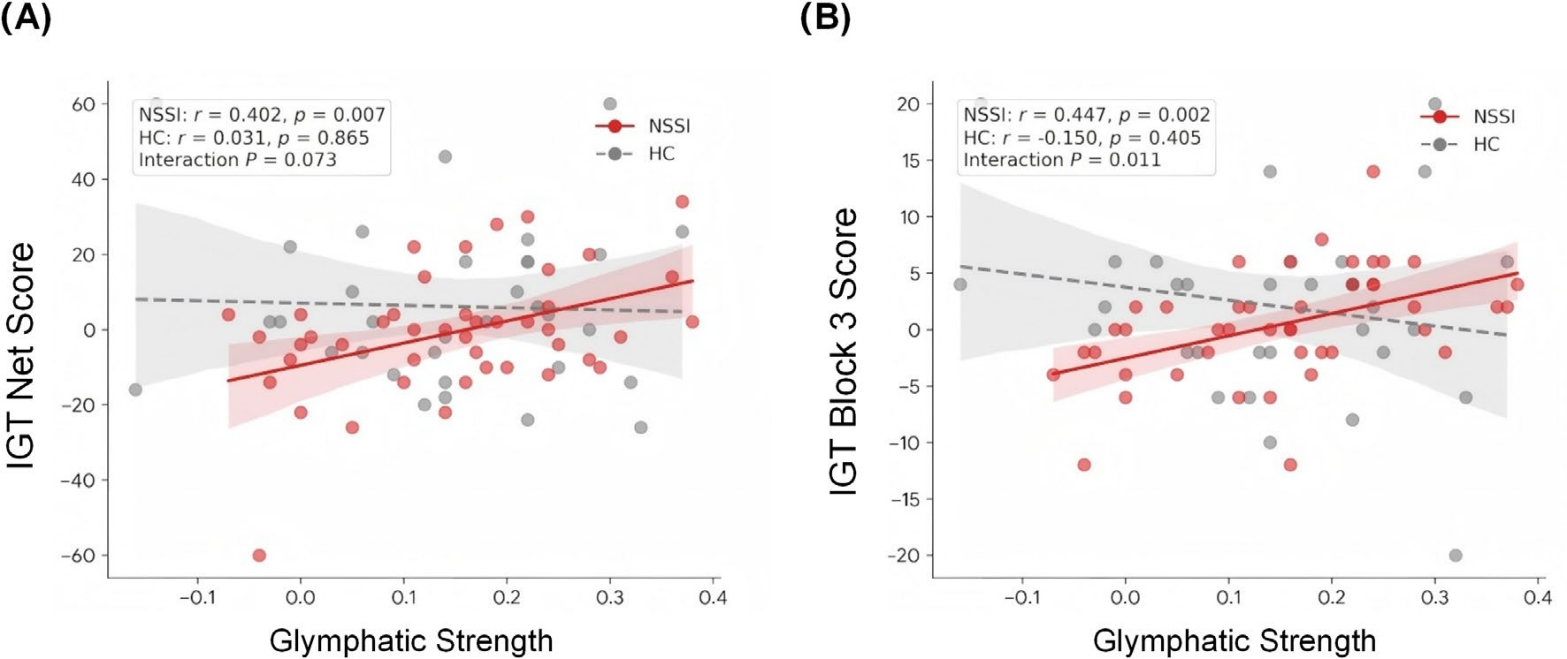
Domain‐ and disease‐specific associations of glymphatic strength with cognitive preservation. Scatter plots show cognitive performance versus glymphatic strength in the NSSI (red, solid lines) and healthy control (HC; gray, dashed lines) groups. Shaded areas indicate 95% confidence intervals. Partial correlation statistics (*r*, *p*) control for age, gender, and mean framewise displacement (FD). (A, B) In the NSSI group, higher glymphatic strength significantly predicted better overall IGT Net Scores (A) and Block 3 Scores (B). HC trends were non‐significant. A significant Group × Coupling interaction in (B) (*p*_interaction_ = 0.011) highlights a disease‐specific compensatory reliance on glymphatic clearance. IGT, Iowa Gambling Task; NSSI, non‐suicidal self‐injury.

### 3.6 Mediating Role of Glymphatic Function in Sleep‐Cognition Pathways

Path analysis within the NSSI cohort revealed that glymphatic strength fully mediated the relationship between subjective sleep quality (PSQI scores) and affective decision‐making (IGT Net Scores; Figure 4). Indirect effect of sleep quality on IGT performance via glymphatic coupling was statistically significant (ab = 0.682), with the bootstrapped 95% confidence interval [0.062, 1.515] strictly excluding zero. Furthermore, the direct effect of PSQI scores on IGT Net Scores was not significant when accounting for this mediator. Crucially, this mediating pathway remained robust and statistically significant even after incorporating baseline depressive severity as an additional covariate in the model (indirect effect = 0.686, bootstrapped 95% CI [0.039, 1.613]; Figure S1C). This confirms that the observed sleep‐glymphatic‐cognition mechanism operates independently of the generalized depressive burden.

**FIGURE 4:**
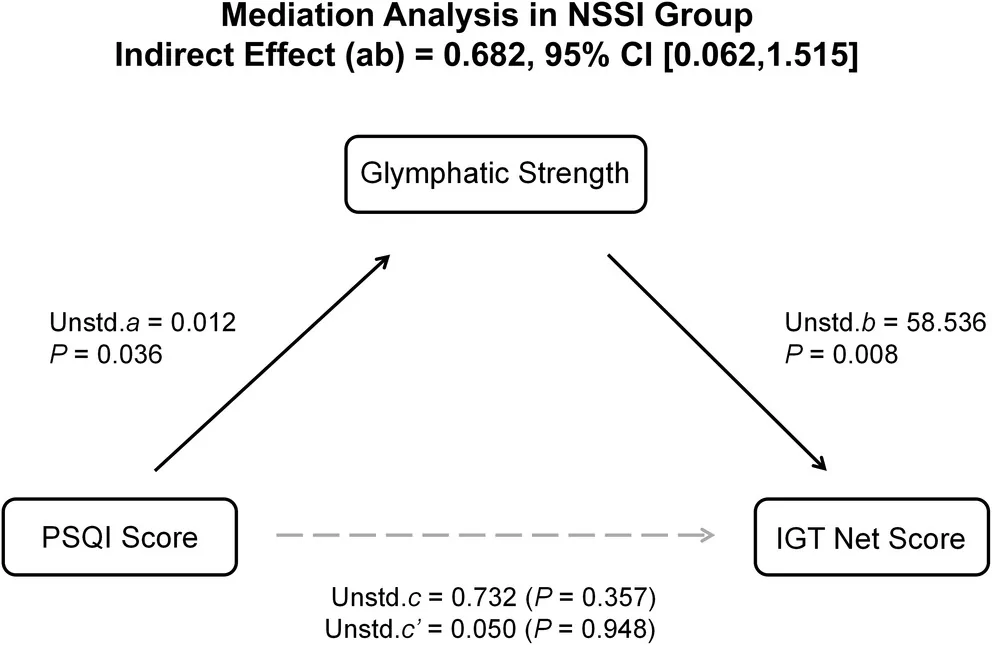
Mediation model of glymphatic strength in the relationship between PSQI score and IGT net score within the NSSI group. Path coefficients shown are unstandardized regression coefficients. Solid arrows represent significant paths, while the dashed gray arrow represents non‐significant paths. Path a indicates the effect of the PSQI score on glymphatic strength; path b indicates the effect of glymphatic strength on the IGT net score. Path c represents the total effect of the PSQI score on the IGT net score without adjusting for the mediator, and path c′ represents the direct effect when the mediator is included in the model. The indirect effect (ab = 0.682) was evaluated using a bootstrapping procedure with 5000 resamples. The 95% confidence interval (CI) for the indirect effect [0.062, 1.515] does not contain zero, indicating a significant full mediation effect. IGT, Iowa Gambling Task; NSSI, non‐suicidal self‐injury; PSQI, Pittsburgh Sleep Quality Index.

## 4 Discussion

This study investigated the macroscopic fluid dynamic mechanisms underlying specific cognitive profiles in adolescents with NSSI. By quantifying resting‐state gBOLD‐CSF coupling, we observed a distinct “clinical‐physiological dissociation.” Our core findings are threefold: (1) In patients with severe subjective sleep disturbances, glymphatic metrics exhibited atypical hyperactivity, which mediated the association between sleep pressure and the preservation of “hot” affective decision‐making; (2) This physiological‐behavioral link was specific to both the affective domain and task phases with high emotion‐cognitive regulation load (IGT Block 3), remaining independent of baseline impulsivity and “cold” executive functions; (3) The predictive value of glymphatic function on strategy execution was unique to the NSSI cohort. Together, these results provide a novel physiological perspective on how the adolescent brain maintains specific network operations under severe psychopathological burden.

A pivotal finding of this study is the mediating role of the glymphatic system between subjective sleep disturbances and the preservation of “hot” affective cognition. While classical models conventionally posit that disrupted sleep architecture impairs glymphatic clearance [20], our data from the NSSI cohort revealed a divergent pattern. Rather than attenuating the coupling signal, exacerbated sleep complaints positively predicted stronger gBOLD‐CSF coupling, which in turn supported sustained IGT performance. This unexpected relationship points toward a stress‐induced compensatory response under pathological conditions.

Evidence from converging lines of research supports this interpretation. Recent neuroimaging work indicates that acute sleep deprivation can trigger a “biphasic adaptation” in fluid dynamics, eliciting a transient compensatory surge in gBOLD‐CSF coupling once a specific sleep pressure threshold is breached [44]. The cumulative sleep burden endured by NSSI patients likely acts as a similar, albeit prolonged, physiological stressor. From a network perspective, this phenomenon aligns with the cortical compensation hypothesis. During sleep deprivation, the brain frequently relies on the hyperactivation of prefrontal or limbic regions to maintain cognitive performance [45]. The atypical enhancement in glymphatic coupling observed here may reflect the clearance response required to offset the heightened metabolic demands of these overworked neural circuits. Furthermore, at the cellular level, microenvironmental shifts driven by chronic stress and waste accumulation can provoke glial responses, potentially modulating AQP4 channels to upregulate fluid clearance [46, 47].

Crucially, the viability of such a robust physiological compensation is likely tethered to the patients' neurodevelopmental stage. In stark contrast to the structural decline of the glymphatic system seen in older adults with neurodegenerative diseases [18, 48], adolescence is characterized by peak neural plasticity and high metabolic capacity [49]. This substantial physiological reserve provides the biological capital necessary to systematically mobilize clearance mechanisms under extreme duress. Consequently, the elevated coupling signature appears to reflect an adaptive response of the young nervous system, striving to manage cumulative metabolic loads and scaffold essential affective decision‐making networks amidst severe clinical deterioration.

The observed behavioral patterns—both within the IGT and between the distinct cognitive paradigms—further suggest that cognitive function in this cohort is not inherently intact, but rather exhibits a “conditional maintenance” contingent upon physiological compensation. During the initial, ambiguous phase of the IGT (Block 1) [50], the NSSI group significantly underperformed compared to HCs. This early behavioral deficit broadly reflects the baseline emotional dysregulation and inhibitory challenges characteristic of the disorder [51]. As the task advanced to the explicit risk and strategy‐formation phase (Block 3), this behavioral gap notably narrowed—a recovery directly correlated with macroscopic clearance metrics [35]. Such a dynamic trajectory implies that the sharply escalating metabolic demands of sustained emotion regulation necessitate enhanced fluid clearance, allowing patients with more robust compensatory coupling to demonstrate greater resilience in suppressing impulsive choices and shifting toward advantageous strategies.

Extending this rationale helps clarify the differential associations observed between “hot” and “cold” cognitive domains. “Hot” affective decision‐making relies heavily on the ventromedial prefrontal cortex (vmPFC) and limbic‐striatal networks [14, 52]—regions frequently characterized by pathological hyperactivity in self‐injuring adolescents [53]. The ensuing local metabolic pressure likely renders the sustained operation of these affective circuits exceptionally dependent on efficient waste clearance [24, 54]. The “cold” executive network, subserved primarily by the dorsolateral prefrontal cortex (dlPFC) [55, 56], operates under different constraints. Experiencing less direct emotional perturbation during neutral cognitive processing, the baseline metabolic demands within the dlPFC might not reach the critical threshold required to trigger brain‐wide compensatory clearance. This neurobiological disparity elegantly explains why the statistical coupling between macroscopic clearance efficiency and cognitive performance emerges selectively under the high metabolic load of “hot” affective processing.

Interaction analyses confirmed the disease‐specificity of this physiological‐behavioral link: glymphatic function predicted strategy execution exclusively in the NSSI group. In healthy brains, baseline metabolic homeostasis easily accommodates routine cognitive challenges [57]. For NSSI adolescents enduring chronic psychopathological attrition, however, the relative efficiency of the glymphatic system becomes a critical factor constraining their vulnerable affective networks. This observation offers a novel perspective for clinical intervention. The macroscopic metabolic clearance mechanism serves not just as a physiological metric, but as a vital buffer sustaining partial cognitive resilience in this vulnerable population. Future clinical strategies might benefit from moving beyond standard symptom management. Exploring interventions that gently optimize sleep architecture (e.g., targeted slow‐wave sleep enhancement) or lifestyle modifications to bolster brain metabolic clearance could prove translationally valuable [43, 58, 59]. Prioritizing the enhancement of underlying physiological compensatory capacities may provide foundational support to protect affective decision‐making networks.

Several methodological limitations warrant consideration. First, the cross‐sectional design of this study inherently limits our findings to correlational associations, precluding definitive causal inferences. Future longitudinal investigations are essential to ascertain whether the observed compensatory upregulation reflects a transient acute stress response or a sustained pathological adaptation. Second, while gBOLD‐CSF coupling provides a robust macroscopic index of global brain clearance [43], it lacks the spatial resolution necessary to directly quantify localized metabolite clearance rates within specific neural micro‐environments. Third, stratifying the cohort into extreme sleep subgroups resulted in relatively small sample sizes. Although the moderate‐to‐large effect size (Cohen's *d* = 0.684) supports the robustness of this physiological divergence, these subgroup findings should be considered preliminary and warrant replication in larger independent cohorts. Fourth, the significant female skew (86.4%) in our NSSI sample accurately reflects the epidemiological profile of the disorder [60, 61], but limits the generalizability of our neurobiological findings to male patients. Subsequent research involving larger, medication‐naive cohorts is warranted to effectively disentangle intrinsic neurovascular dynamics from pharmacological effects. Finally, the potential for undetected transient sleep remains a limitation, even with continuous video tracking of participant wakefulness. Resolving this requires future studies to utilize fast‐fMRI coupled with simultaneous pupillometry or EEG/EOG. By capturing objective arousal states, these multimodal approaches could help untangle the underlying neural and physiological drivers governing the observed macroscopic fluid coupling alterations [62, 63, 64].

In summary, this study highlights the glymphatic system as a crucial compensatory buffer that scaffolds specific cognitive functions in adolescents with comorbid MDD and NSSI. Confronted with the dual burdens of cumulative sleep disturbances and severe psychopathological stress, the developing nervous system appears to systematically mobilize its physiological reserves to meet the heightened metabolic demands of affective decision‐making networks. Beyond advancing our pathophysiological understanding, these findings provide compelling neuroimaging evidence for the brain's dynamic capacity to orchestrate physiological compensation under extreme psychiatric duress, ultimately pointing toward novel neurometabolic targets for clinical intervention.

## Funding

This work was supported by the National Natural Science Foundation of China (Grant Nos. U24A20702 and 32300869), Anhui Provincial Natural Science Foundation (Grant No. 2308085QH267).

## Ethics Statement

The study was approved by the Ethics Committee of Anhui Medical University and was conducted in accordance with the Declaration of Helsinki (approval ethics numbers: 83230422).

## Conflicts of Interest

The authors declare no conflicts of interest.

## Supporting information


**Table S1:** Distribution of NSSI frequency in the NSSI group.
**Table S2:** Comparison of BIS‐11 scores between the NSSI and HC groups.
**Table S3:** Medication information for the NSSI patients.
**Table S4:** Comprehensive comparison of cognitive sub‐dimensions between the NSSI and HC groups.
**Table S5:** Correlations between glymphatic strength and clinical variables in NSSI.
**Table S6:** Associations between glymphatic function and cognitive performance in the NSSI group.
**Figure S1:** Robustness of the physiological‐behavioral mechanism after controlling for depressive severity.
**Figure S2:** Moderation analysis of age on the relationship between glymphatic strength and affective decision‐making.

## Data Availability

The data that support the findings of this study are available from the corresponding author upon reasonable request.
