# Supplementary material for: The Role of Glymphatic Clearance in Affective Decision‐Making in Adolescents with Non‐Suicidal Self‐Injury
Source: CNS Neurosci Ther. 2026 Oct 1;32(10):e71159. doi: 10.1002/cns.71159 (PMC13628287; doi:10.1002/cns.71159)
Supplement: Supplementary file 1 [file CNS-32-e71159-s001.docx]

**Supplementary Materials**

**List of Supplementary Contents**

- **Table S1.** Distribution of NSSI frequency in the NSSI group.
- **Table S2.** Comparison of BIS-11 scores between the NSSI and HC groups.
- **Table S3.** Medication information for the NSSI patients.
- **Table S4.** Comprehensive comparison of cognitive sub-dimensions between the NSSI and HC groups.
- **Table S5.** Correlations between glymphatic strength and clinical variables in NSSI.
- **Table S6.** Associations between glymphatic function and cognitive performance in the NSSI group.
- **Figure S1.** Robustness of the physiological-behavioral mechanism after controlling for depressive severity.
- **Figure S2.** Moderation analysis of age on the relationship between glymphatic strength and affective decision-making.

**Table S1. Distribution of NSSI frequency in the NSSI group (n=44).**

| NSSI Frequency (Past Year) | n (%) |
| --- | --- |
| 1–5 acts | 2 (4.5%) |
| 6–10 acts | 2 (4.5%) |
| 11–20 acts | 9 (20.5%) |
| 21+ acts | 31 (70.5%) |
| Total | 44 (100%) |

Note: Data are presented as number of participants (n) and percentage (%). NSSI frequency refers to the number of self-injury acts reported in the past 12 months.

Abbreviations: NSSI, Non-suicidal self-injury.

**Table S2. Comparison of BIS-11 scores between the NSSI and HC groups.**

| BIS Variable | NSSI(n = 44) | HC(n = 33) | Statistic(*t*) | *P*-value |
| --- | --- | --- | --- | --- |
| Motor Impulsiveness | 50.80 ± 14.79 | 32.59 ± 12.12 | 5.93 | < 0.001*** |
| Cognitive Impulsiveness | 49.70 ± 14.95 | 45.98 ± 12.05 | 1.21 | 0.231 |
| Non-planning Impulsiveness | 63.57 ± 14.43 | 48.39 ± 12.45 | 4.94 | < 0.001*** |
| Total Score | 54.69 ± 11.75 | 42.32 ± 8.57 | 5.34 | < 0.001*** |

Note: *t* values were calculated using independent samples *t*-tests. Data are presented as mean ± SD. ****P* < 0.001. Abbreviations: BIS-11: Barratt Impulsiveness Scale-11; NSSI, Non-suicidal self-injury; HC: Healthy control; SD: Standard deviation.

**Table S3. Medication information for the NSSI patients.**

| Characteristic | n (%) |
| --- | --- |
| Medication Status | |
| Medication-naive | 20 (45.50%) |
| Medicated | 24 (54.50%) |
| Medication Regimen (among medicated, n=24) | |
| Monotherapy | 8 (33.30%) |
| Polypharmacy | 16 (66.70%) |
| Psychotropic Drug Classes (among medicated, n=24) | |
| SSRIs | 23 (95.80%) |
| SNRIs | 2 (8.30%) |
| Antipsychotics | 12 (50.00%) |
| Anxiolytics / Sedatives | 4 (16.70%) |

Note: Data are presented as number of participants (n) and percentage (%). Percentages for medication status are calculated based on the total sample (n = 44). Percentages for medication regimen and drug classes are calculated based on the medicated subgroup (n = 24). Monotherapy refers to the use of a single psychotropic agent; Polypharmacy refers to the concurrent use of two or more psychotropic agents. Categories of drug classes are not mutually exclusive as some patients received multiple types of medications. Abbreviations: NSSI, Non-suicidal self-injury; SSRIs: Selective Serotonin Reuptake Inhibitors; SNRIs: Serotonin-Norepinephrine Reuptake Inhibitors.

**Table S4. Comprehensive comparison of cognitive sub-dimensions between the NSSI and HC groups.**

| Cognitive Domain | NSSI(n = 44) | HC(n = 33) | Statistic(Z/*t*) | *P*-value |
| --- | --- | --- | --- | --- |
| Iowa Gambling Task (IGT) | | | | |
| Block1 | -4(-6, 0) | -2(-4, 2) | -2.29 | 0.022* |
| Block2 | -2(-6, 2) | -2(-4, 4) | 0.00 | 1.000 |
| Block3 | 0(-2, 2) | 0(-2, 6) | -1.13 | 0.260 |
| Block4 | 2(-2, 6) | 2(-2, 6) | -0.19 | 0.848 |
| Block5 | 0(-4, 4) | 2(-2, 8) | -0.44 | 0.659 |
| Wisconsin Card Sorting Test (WCST) | | | | |
| Categories Completed | 13.5(9, 22.25) | 12(8, 18) | -0.62 | 0.533 |
| Perseverative Errors | 15(10, 19) | 16(11, 21) | -0.52 | 0.606 |
| Non-Perseverative Errors | 1(0, 2) | 1(0, 1) | -0.55 | 0.585 |
| Unique Errors | 6(4, 6) | 6(5, 6) | -0.70 | 0.484 |

Note: Continuous variables were tested using Mann-Whitney *U* tests due to non-normal distributions, and are presented as median (25th percentile, 75th percentile). **P* < 0.05. Abbreviations: NSSI, Non-suicidal self-injury; HC: Healthy control.

**Table S5. Correlations between glymphatic strength and clinical variables in NSSI.**

| Clinical Variable | *r_partial_* | *P-value* | *r* | *P-*value^a^ |
| --- | --- | --- | --- | --- |
| HAMD | -0.029 | 0.854 | 0.041 | 0.792 |
| BIS-11 | 0.003 | 0.983 | -0.018 | 0.907 |
| CRSNSSI grade | 0.009 | 0.952 | 0.034 | 0.828 |

Note: Unadjusted Model represents raw correlation coefficients (Pearson *r* for continuous variables, Spearman *ρ* for ordinal variables) between glymphatic strength and clinical variables. Adjusted Model represents partial Pearson correlation coefficients (*r_partial_*) controlling for age, gender, and mean framewise displacement (FD). ^a^Uncorrected *P*-values are reported. **P* < 0.05. Abbreviations: NSSI, Non-suicidal self-injury; HAMD, Hamilton Depression Rating Scale; BIS-11, Barratt Impulsiveness Scale-11; HAMA, Hamilton Anxiety Rating Scale; CRSNSSI, Clinician-Rated Severity of Non-Suicidal Self-Injury.

**Table S6.** **Associations between glymphatic function and cognitive performance in NSSI.**

| Cognitive Domain | *r_partial_* | *P-*value | *P_FDR_* |
| --- | --- | --- | --- |
| Iowa Gambling Task (IGT) | | |  |
| Net Score (Total) | 0.402 | 0.007* | 0.032* |
| Block1 | 0.203 | 0.187 | 0.262 |
| Block2 | 0.232 | 0.131 | 0.236 |
| Block3 | 0.447 | 0.002* | 0.018* |
| Block4 | 0.061 | 0.694 | 0.694 |
| Block5 | 0.266 | 0.081 | 0.182 |
| Wisconsin Card Sorting Test (WCST) | | |  |
| Total Errors | 0.270 | 0.076 | 0.182 |
| Perseverative Errors | 0.195 | 0.204 | 0.262 |
| Categories Completed | -0.163 | 0.291 | 0.327 |

Partial correlations controlling for age, sex, mean FD. **P* < 0.05.

*P_FDR_* represents *P*-values corrected for multiple comparisons using the Benjamini-Hochberg False Discovery Rate procedure.

Abbreviations: NSSI, Non-suicidal self-injury; IGT, Iowa Gambling Task; WCST, Wisconsin Card Sorting Test.

**Figure S1. Robustness of the physiological-behavioral mechanism after controlling for depressive severity.**

(A) Partial correlation scatter plot demonstrating that the positive association between subjective sleep quality (PSQI) and glymphatic strength remains robust after strictly controlling for HAMD-17 scores (*r_partial_* = 0.321, *P* = 0.034). (B) Partial correlation scatter plot illustrating that higher glymphatic strength continues to significantly predict better affective decision-making (IGT Net Score) when controlling for HAMD-17 (*r_partial_* = 0.402, *P* = 0.007). In A and B, the axes represent the residuals of the variables after regressing out the respective covariates, with shaded areas indicating 95% confidence intervals. (C) The full mediation model incorporating HAMD score as a covariate. Path coefficients shown are unstandardized (Unstd.) regression coefficients. The indirect effect of sleep quality on IGT performance via glymphatic clearance remains statistically significant (ab = 0.686, 95% CI [0.039, 1.613]), confirming that this compensatory mechanism operates independently of the patients’ generalized depressive symptoms. Abbreviations: NSSI, Non-suicidal self-injury; IGT, Iowa Gambling Task; HAMD, Hamilton Depression Rating Scale; PSQI, Pittsburgh Sleep Quality Index.

**Figure S2. Moderation analysis of age on the relationship between glymphatic strength and affective decision-making.**

(A) Conceptual moderation model. The diagram illustrates the hypothesis that age (W) acts as a moderator on the direct pathway from baseline glymphatic strength (X) to the Iowa Gambling Task (IGT) net score (Y). The calculated unstandardized path coefficients (b) and P-values are presented. The interaction term was not statistically significant (b3 = 2.88, P = 0.769), indicating the absence of a moderation effect.

(B) Simple slopes plot. The scatter plot with regression lines visualizes the relationship between glymphatic strength and IGT net scores across different age levels (Mean Age, -1 SD, and +1 SD) within the NSSI cohort. The nearly parallel lines intuitively confirm that this compensatory physiological-behavioral coupling is remarkably stable and not moderated by ongoing neurodevelopmental changes during adolescence. The ordinary least squares (OLS) regression analysis employed mean-centered predictors and strictly controlled for gender and mean framewise displacement.
